# Totally Endoscopic Replacement of the Ascending Aorta and the Aortic Root including the Aortic Valve via Right Mini-Thoracotomy: A Multicenter Study

**DOI:** 10.3390/jcm13092648

**Published:** 2024-04-30

**Authors:** Marwan Hamiko, Saad Salamate, Maedeh Ayay Nassari, Andre Spaeth, Sami Sirat, Mirko Doss, Mohamed Amer, Miriam Silaschi, Ali El-Sayed Ahmad, Farhad Bakhtiary

**Affiliations:** 1 Department of Cardiac Surgery, University Hospital Bonn, Venusberg Campus 1, 53127 Bonn, Germany; saad.salamate@ukbonn.de (S.S.); maedehnassari@yahoo.com (M.A.N.); andre.spaeth@ukbonn.de (A.S.); miriam.silaschi@ukbonn.de (M.S.); ali.assayed@googlemail.de (A.E.-S.A.); farhad.bakhtiary@ukbonn.de (F.B.); 2 Department of Cardiac Surgery, Helios Hospital Siegburg, 53721 Siegburg, Germany; abdul-sami.sirat@helios-gesundheit.de (S.S.); mirko.doss@helios-gesundheit.de (M.D.); 3 Department of Cardiac Surgery, Helios Hospital Wuppertal, 42283 Wuppertal, Germany; mohamedishaq2000@yahoo.com

**Keywords:** right mini-thoracotomy, endoscopic surgery, minimally invasive aortic replacement, minimally invasive aortic root replacement

## Abstract

**Background**: Recently, minimally invasive access via right anterolateral mini-thoracotomy (RAMT) has been gaining popularity in cardiac surgery. This approach is also an option for surgeons performing aortic surgery. The aim of this study is to present our surgical method, highlighting the total endoscopic minimally invasive approach via RAMT for replacement of the ascending aorta (AAR) with or without involvement of the aortic root and the aortic valve. **Methods**: Clinical data of 44 patients from three participating institutions with AAR with or without involvement of the aortic valve or aortic root via RAMT between April 2017 and February 2024 were retrospectively analyzed. According to surgical procedure, patients were divided into two groups, in the AAR and in the Wheat/Bentall group with concomitant valve or root replacement. Operative time, length of ventilation, perioperative outcome, length of intensive care unit (ICU) as well as postoperative hospital stay, and mid- and long-term results were retrospectively analyzed. **Results**: Mean age was 61.4 ± 10.7 years old with a frequency of male gender of 63.6%. Mean cardiopulmonary bypass (CBP) time and aortic cross-clamping time was 94.9 ± 32.5 min and 63.8 ± 25.9 min, respectively. CPB and aortic clamp time were significantly lower in AAR group. In the first 24 h, the mean drainage volume was 790.3 ± 423.6 mL. Re-thoracotomy due to bleeding was zero. Sternotomy was able to be avoided in all patients. Patients stayed 35.9 ± 23.5 h at ICU and were discharged 7.8 ± 3.0 days following surgery from hospital. Mean ventilation time was 5.8 ± 7.6 h. All patients survived and 30-day mortality was 0.0%. At a median follow-up time of 18.2 months, all patients were alive. The results were similar in both groups. **Conclusions**: The full endoscopic RAMT approach with 3D visualization is a safe, feasible and promising technique that can be transferred in the field of aortic surgery without compromising surgical quality, postoperative outcomes, or patient safety when performed by an experienced team in a high-volume center.

## 1. Introduction

Since the late 1990s, minimally invasive surgery via right anterolateral mini-thoracotomy (RAMT) access has been increasing and gaining popularity in cardiac surgery in order to optimize surgical procedures and results. Actually, minimally invasive surgery via RAMT has become established in many centers worldwide in the field of valve surgery [1,2,3,4,5,6,7,8,9,10,11,12]. The standard surgical approach for the treatment of pathologies of the ascending aorta (AA) and the aortic root is the complete median sternotomy [13,14]. However, several studies report excellent results using a minimally invasive upper partial sternotomy for treating pathologies of the AA and of the aortic root [15,16,17,18,19,20]. In order to reduce surgical trauma and sternotomy-associated complications, many cardiac surgeons switch to RAMT access to treat AA and aortic root pathologies. However, only a few studies with limited selected patients present the outcomes of this approach with favorable results [21,22,23,24,25]. At our institution, the RAMT approach has been standardized as a well-established minimally invasive approach for valve surgery as well as for aortic surgical procedures including the aortic root and the aortic valve.

The aim of this study is to describe the steps of our surgical approach in replacement of the AA (AAR) with or without involvement of the aortic root and the aortic valve via RAMT in full endoscopic technique using 3D visualization. Moreover, we present the mid- and long-term outcomes.

## 2. Materials and Methods

### 2.1. Study Population

After approval by the ethics committee of the University of Bonn (ethical approval No. 464/22) and written informed consent from all participating patients, clinical data of 44 patients aged over 18 years from three participating institutions who underwent elective supracoronary AAR, Wheat’s or Bentall’s procedure via RAMT between April 2017 and February 2024 were retrospectively analyzed. All enrolled patients suffered from an aneurysm of the AA or the aortic root with concomitant aortic valve stenosis or regurgitation documented by computed tomography (CT) and echocardiography. Two patients had an aortic diameter < 50 mm and were scheduled for isolated replacement of the ascending aorta. One patient with an aortic diameter of 49 mm had a progression of 4 mm during a follow-up of 6 months; the other patient with an aortic diameter of 47 mm had a bicuspid aortic valve without stenosis or insufficiency, but the patient had a family history of aneurysmal disease and was psychologically burdened by the diagnosis, so the patient was also scheduled for the surgery as per his wishes. Patients with acute or chronic aortic dissection, redo cardiac surgery, endocarditis patients, and patients scheduled for the David procedure were excluded from the study.

A comprehensive data set of pre-, intra-, and postoperative parameters was analyzed by review of institutional databases. Baseline characteristics, including cardiovascular risk factors, former cerebrovascular disease, comorbidities, presenting symptoms by classifying in NYHA-classification as well as echocardiographic and CT data were calculated and recorded.

### 2.2. Study Groups

According to the performed procedures, patients were divided into two groups:

- AAR: Supracoronary AAR, *n* = 14;
- Wheat/Bentall: Wheat’s procedure (supracoronary AAR concomitant AVR) or Bentall’s procedure (replacement of the aortic root and AVR with a conduit), *n* = 30.

### 2.3. Preoperative Diagnostic Tools

Every patient was scheduled for surgery after finishing the necessary preoperative diagnostics. This included echocardiography to assess the pathology of the aortic valve, coronary angiography to exclude coronary artery disease, regular pulmonary function testing, and CT scan to assess the anatomy of the whole aorta, including the aortic root and the groin vessels as an access for cardiopulmonary bypass (CBP). In the case of calcified groin vessels or abdominal aorta, we usually use the axillary artery as an access for CPB.

### 2.4. Indication for Surgery

According to the EACT/STS guidelines, patients with an aneurysm of the AA or the aortic root with a measured diameter of >55 mm or with rapid progression of the aneurysm during follow-up were scheduled for surgery [26]. In patients with an indication for AVR, concomitant AAR is recommended if the diameter reaches 45 mm [26]. In the case of aortic root involvement and the David procedure not being possible, patients received the Bentall procedure; otherwise, a Wheat procedure was performed in the case of normal diameter of the aortic root.

### 2.5. Surgical Procedure

**Figure 1:**
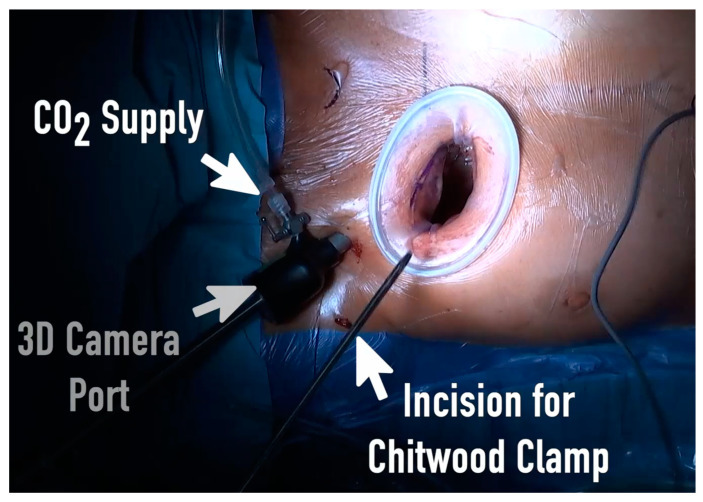
View of the surgical field with right anterior mini-thoracotomy (RAMT) via the third intercostal space with the soft tissue retractor, the 3D camera port, the incision for the Chitwood aortic clamp, and the CO_2_ supply.

A standard anesthetic protocol was performed using sufentanil, propofol, and rocuronium for induction of general anesthesia followed by single-lumen endotracheal intubation. Anesthesia was maintained using sufentanil and sevoflurane and was continued during CPB. Routinely, standard monitoring including electrocardiogram, invasive blood pressure, near-infrared spectroscopy (NIRS), and transesophageal echocardiography (TEE) were applied. Every patient had external defibrillation electrode pads placed behind the right scapula and on the lateral side of the left anterior axillary line. Anticoagulation with 400–500 U/kg sodium heparin was initiated to achieve an activating clotting time of >450 s. After sterile washing and draping of the patient, femoral venous and arterial cannulation was performed percutaneously using the Seldinger technique and under TEE control to conduct the CPB (Terumo Cardiovascular Systems, Ann Arbor, MI, USA). In the case of calcified groin vessels or abdominal aorta, the arterial cannulation was performed via axillary artery. Standard size of arterial cannula was 16–8Fr (Bio-Medicus arterial cannulas; Medtronic, Minneapolis, MN, USA); the femoral vein was cannulated with 25–27Fr multistage cannula (Bio-Medicus arterial cannulas; Medtronic, Minneapolis, MN, USA) with placement in the superior vena cava. In patients with a body weight more than 80 kg, an additional cannula was placed via the right jugular vein in the superior vena cava. After establishment of the CBP, the RAMT approach was performed via a small skin incision of 3–5 cm in the second or third intercostal space (ICS). Afterward, the pleural space was entered without transection of the mammary vessels or resection of the rips. Extension of the ICS was performed by insertion of the soft tissue retractor (Valve GateTM Soft Tissue Protector, Geister, Germany). The use of a rip spreader for further visualization was avoided. The whole surgical procedure was performed via a totally endoscopic approach in 3D visualization. Two further small incisions of 5 mm were made in the second ICS for the 3D camera port (Aesculap EinsteinVision, Tuttlingen, Germany) and for aortic cross-clamping (Figure 1).

The pericardium was opened while sparing the phrenic nerve and sutures for pericardial stay were placed to provide sufficient exposure. In the case of sufficient aortic valve, a needle vent catheter was placed in the AA. A left ventricular vent catheter was placed in the right superior pulmonary vein in all cases. Transincisional direct aortic cross-clamping was performed using a Chitwood cross-clamp (Scanlan International, Inc. St. Paul, MN, USA). The carbon dioxide (CO_2_) diffuser was connected to the camera port. Myocardial protection was achieved using Brettschneider cardioplegia (Custadiol, Dr. Franz Kohler Chemie GmbH, Bensheim, Germany) via the inserted needle vent catheter. In the case of aortic valve regurgitation, cardioplegic solution was infused directly in both coronary ostia after the aortotomy. A non-pulsatile pump flow of 2.2–2.6 L/min/m^2^ was conducted to maintain a mean arterial pressure (MAP) of 50–60 mm Hg during CPB. Based on aortic pathology, the proximal AA was resected. If the aneurysm was localized only to the AA, a supracoronary AAR was performed. In the case of additional aortic valve pathology (aortic valve stenosis or regurgitation) with or without involvement of the aortic root, either a Wheat procedure or the Bentall procedure was performed.

#### 2.5.1. Supracoronary Replacement of the Ascending Aorta

**Figure 2:**
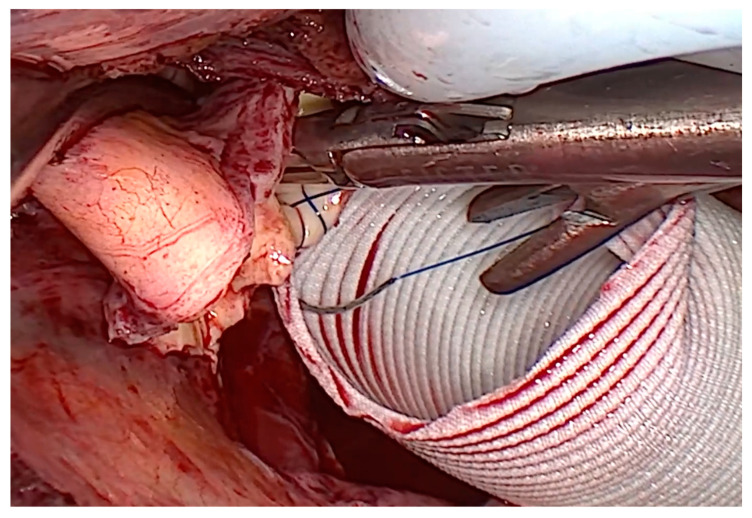
Surgical view from the 3D camera during completion of the distal anastomosis without the need for circulatory arrest.

After removing the AA, first the distal then the proximal anastomosis between the distal and proximal part of the native aorta and the suitable vascular graft, with usual size between 28 mm and 34 mm (Vascutek Gelweave grafts, Vascutek Terumo Inc., Inchinnan, Renfrewhire, Scotland, UK), was performed in a running fashion using a 4–0 polypropylene suture (Figure 2). The length of the prosthesis was always chosen according to the surgeon’s experience. After resection of the aneurysm and finishing the proximal or distal anastomosis, the length of the prosthesis was determined in situ.

#### 2.5.2. Wheat Procedure (Video Included as Supplementary Material)

**Figure 3:**
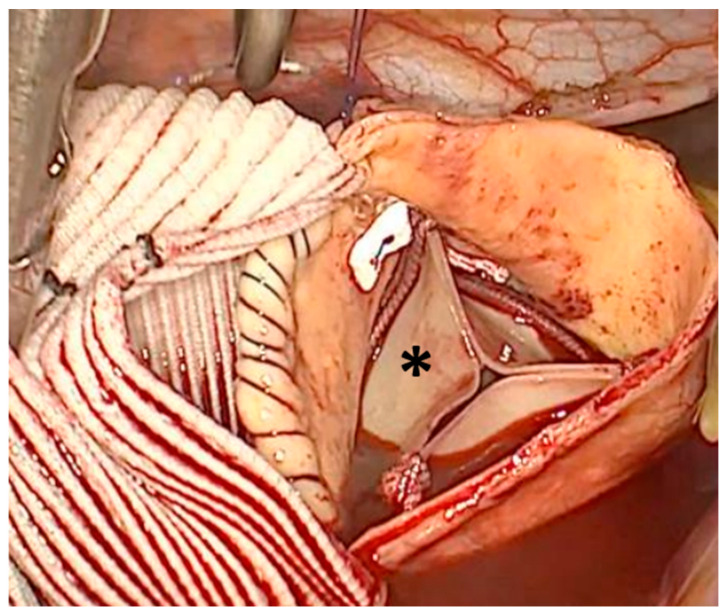
Surgical view from the 3D camera during completion of the proximal anastomosis after finishing the aortic valve replacement with biological prosthesis (*).

After removing the AA, polypropylene stay sutures were placed to exposure the aortic valve. The leaflets were excised, and in the case of calcified valves, a debridement of the annulus was performed. According to actual ESC/EACTs guidelines and patients’ wishes, biological aortic valves were implanted in all patients [27]. The size of the prosthesis was based on intraoperative measurement using the according valve sizer. For AVR, pledgeted mattress annular sutures were placed using the automatically RAM^®^suture device (RAM^®^ COR-SUTURE^®^ QUICK LOAD^®^, LSI Solutions, Victor, NY, USA) starting with the base of the non-coronary cusp, followed by the left coronary cusp, and finally the right coronary cusp, respectively. Before the introduction of the RAM^®^ suture device (RAM^®^ COR-SUTURE^®^ QUICK LOAD^®^, LSI Solutions, Victor, NY, USA) into our routine practice, annular sutures were traditionally placed manually using interrupted non-everted pledgeted mattress sutures, starting at the base of the left coronary cusp and proceeding in a clockwise fashion. An automatic knot-tie device (Cor-Knot^®^, LSI Solutions, Victor, NY, USA) was used to tie the knots of the aortic valve prosthesis. After finishing the AVR, the supracoronary AA was replaced using the same technique described above (Figure 3).

#### 2.5.3. Bentall Procedure

After performing the aortotomy, the complete AA, including the aortic root, was resected. The coronary buttons were cut out and retracted using stay sutures, and the remaining portion of aortic sinuses was removed. Afterwards, the leaflets of the aortic valve were removed. After sizing the aortic annulus, a suitable biological conduit (size of vascular graft = 5 mm > size of aortic valve prosthesis) was constructed, whereas a vascular graft (Vascutek Gelweave grafts, Vascutek Terumo Inc., Scotland, UK) and a bioprosthetic valve were sewn into the graft using a running 3–0 polypropylene. Then, pledgeted mattress annular sutures were placed manually either using interrupted non-everted pledgeted mattress sutures, starting at the base of the left coronary cusp and proceeding in a clockwise fashion, or using the automatic RAM^®^ suture device (RAM^®^ COR-SUTURE^®^ QUICK LOAD^®^, LSI Solutions, Victor, NY, USA), starting with the base of the non-coronary cusp, followed by the left coronary cusp, and finally the right coronary cusp, respectively. The automatically knot tie device (Cor-Knot^®^, LSI Solutions, Victor, NY, USA) was used to tie the sutures with the constructed conduit. Further sutures were placed from aortic side and are also tied with the conduit to stabilize the conduit with the aortic root. Thereafter, both coronary buttons (starting with the left main artery) were anastomosed on the vascular graft using 5-0 polypropylene sutures. After determination of the length of the vascular graft the distal anastomosis between the graft and the remaining aorta was completed using 4–0 polypropylene suture.

After completing the surgical procedure and before de-clamping the aorta, temporary epicardial pacing wire was positioned on the right ventricle. Before releasing the aortic cross-clamp, the left ventricle and the vascular graft were de-aired under TEE guidance using an aortic de-airing cannula and the left ventricular vent. TEE was also used to evaluate aortic prosthesis function, global ventricle functions, and blood flow of the coronary arteries. CPB was ended, and the vein cannula was removed. During protamine administration and obtaining further hemostasis, the arterial cannula was removed and the femoral artery was closed using the MANTATM closure system (Essential Medical Inc., Malvern, PA, USA). A pleural and a pericardial tube were placed, respectively. All incisions were closed in the usual fashion.

### 2.6. Postoperative Care

After the surgical procedure was finished, all patients were transferred to the intensive care unit (ICU), where early extubation and transfer to the intermediate care (IMC) ward were attempted. In some cases, the patients were extubated in the operating room (fast track). After arrival at the ICU, blood loss from chest tube drainages was monitored after 1, 2, 3, 6, and 24 h postoperatively. In addition, transfusion of red blood cells (RBC), fresh frozen plasma (FFP), and platelet concentrates (PC) during surgery and ICU stay were evaluated, as well as any additional surgery or adverse event during the hospital stay. According to institutional policy, bleeding was defined as a mean blood loss exceeding 400 mL/h measured between arrival at ICU and the earliest of the following events: the elapse of 3 h or re-thoracotomy due to major blood loss, as generally accepted.

Intra- and postoperative data, including operative time based on aortic cross-clamping time and duration of the CPB, as well as postoperative outcome, including length of ventilation, length of ICU, and postoperative hospital stay, re-thoracotomy due to bleeding, requirement of blood transfusion, and in-hospital and 30-day mortality were also analyzed.

### 2.7. Follow-Up Data

During follow-up, all patients were regularly contacted at 1, 3, and 6 months and at 1, 3, and 5 years postoperatively. Follow-up data were obtained through telephone contact and included questions on survival and aortic reintervention.

### 2.8. Statistical Analysis

Statistical analysis was performed using the IBM SPSS statistics version 25 (SPSS Inc., Chicago, IL, USA) and GraphPad Prism version 8.4.3 (LaJolla). Continuous variables were expressed as mean ± standard deviation (SD), and categorical variables were given as absolute values and percentages. Data were tested for normal distribution by using Kolmogorov–Smirnov and Shapiro–Wilk tests. Normally distributed demographic and clinical data were analyzed using Student’s *t*-test. Not normally distributed data were compared using the Mann–Whitney U-test. Categorial variables were evaluated with the Pearson chi-square test or Fisher’s exact test, as indicated. A *p*-value of <0.05 was considered statistically significant.

## 3. Results

Baseline characteristics, aortic valve pathologies, comorbidities, symptoms at admission, and preoperative CT and echocardiographic parameters are summarized in Table 1. In total, 44 patients were included in the actual study (14 patients with supracoronary AAR and 30 patients with the Wheat or Bentall procedure). Table S1 shows how many patients were operated on in which center and which procedures were performed. In total, there were 28 (63.6%) male and 16 (36.4%) female patients with a mean age of 61.4 ± 10.7 years. In total, 41 (93.2%) patients suffered from hypertension. A medical history of cardiac artery disease (CAD) was present in 18.2% of the study population. Previous stroke without neurological deficit was recorded in 9.1% of the patients. Nearly half of the patients had bicuspid aortic valves, and 6.8% had a bovine arch configuration. In patients who needed AVR, aortic regurgitation and aortic stenosis were present in 14 (31.8%) and 16 (36.4%) patients, respectively.

Most of the patients presented with dyspnea according to the NYHA class II and III. The preoperative echocardiographic data revealed normal LVEF in both groups. The mean aortic diameter, measured in the CT scans, was 51.2 ± 4.6 mm at the level of the ascending aorta. The range of the diameter was 47–66 mm in the AAR group and 45–64 mm in the Wheat/Bentall group, respectively.

Regarding the previous medical history, no differences were documented between both groups.

In Table 2, the intraoperative data and the postoperative outcome are presented. Adequate surgical exposure without the need for conversion to sternotomy was obtained in all patients. Mean procedure time was 140.6 ± 33.2 min; mean aortic cross-clamp time was 63.8 ± 25.9 min, whereas the mean CBP time was 94.9 ± 32.5 min. None of the patients needed circulatory arrest for performing the distal anastomosis. The intraoperative transfusion rate of RBC and FFP was in total very low.

**Figure 4:**
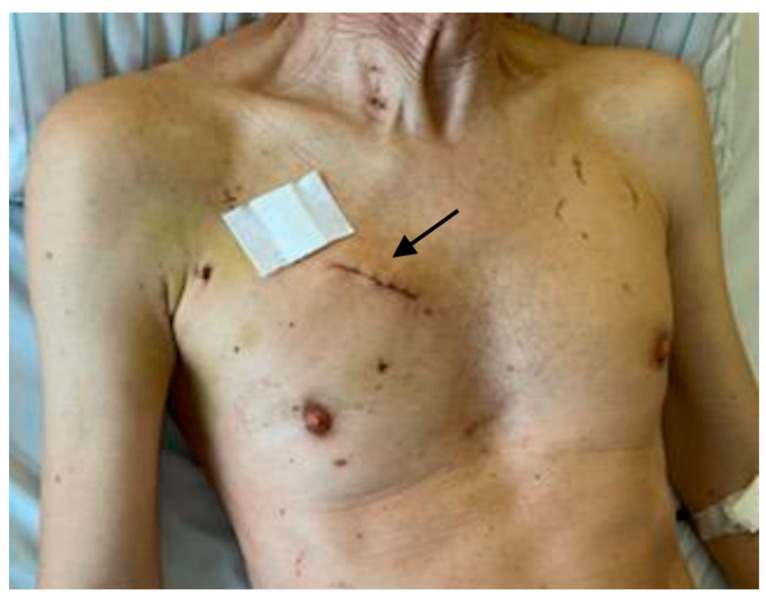
Postoperative view of the RAMT approach with the cosmetic result of the skin incision (black arrow).

There was no significant postoperative complication noted, including bleeding, pericardial effusion needing re-thoracotomy, myocardial infarction, conversion to sternotomy, renal failure, postoperative stroke, or in-hospital deaths. Postoperative delirium was present in 9.1% of the entire cohort. The rate of postoperative atrial fibrillation was 20.5%. Requirement of postoperative transfusion was also very low. None of the patients needed pacemaker implantation. The rate of wound infection was zero. The cosmetic result was excellent (Figure 4).

Mean mechanical ventilation time was 5.8 h. Patients stayed nearly 36 h at the ICU and were discharged after a mean stay of 7.8 ± 3.0 days. In-hospital, 30-day, and one-year mortality was zero, respectively. Throughout the midterm follow-up period of 18.2 months (range: 1–62 months), no occurrences of valve deterioration, cardiovascular events necessitating reintervention, or mortality were observed.

**Figure 5:**
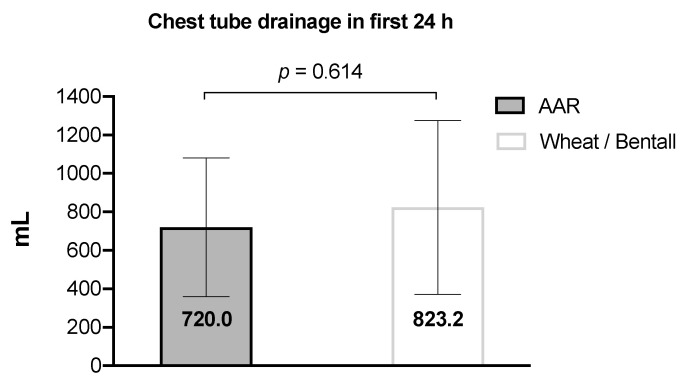
Postoperative bleeding amount in the first 24 h. Data are presented as mean with SD in columns. Abbreviation: AAR—replacement of the ascending aorta.

Figure 5 shows the recorded amount of postoperative bleeding from the chest tube drainage with a mean loss of 790.3 ± 423.6 mL during the first 24 h postoperatively.

Regarding the procedure time, aortic cross-clamp time, and CBP time, these were significantly lower in patients of AAR (AAR: 121.0 ± 27.1, 42.9 ± 19.4, and 68.8 ± 19.7 min vs. Wheat/Bentall: 149.8 ± 32.2, 73.5 ± 22.7, and 107.2 ± 30.0 min; *p* < 0.05, respectively.

**Figure 6:**
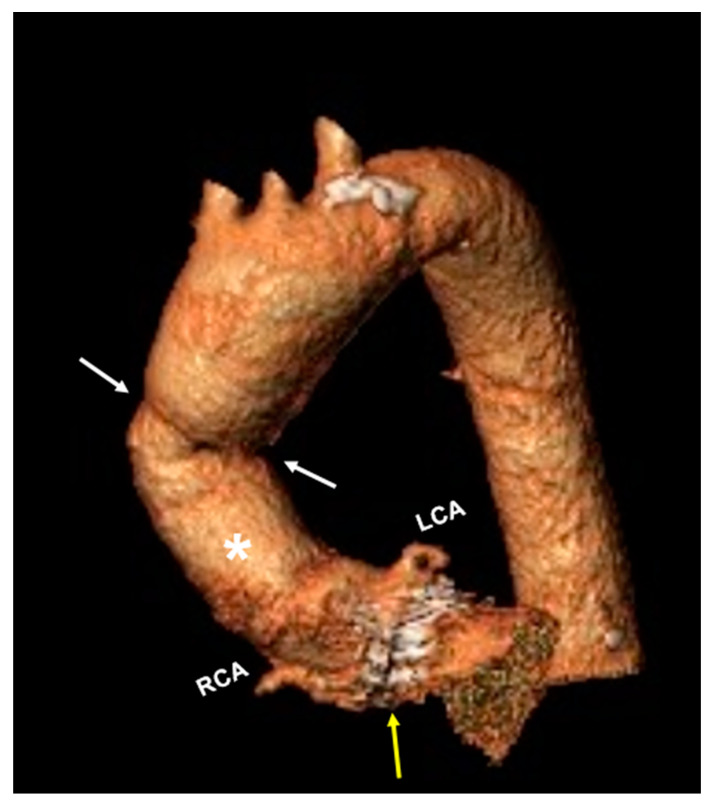
Postoperative CTA examination and three-dimensional reconstruction indicated a successful Bentall procedure with replacement of the ascending aorta (white asterisk), the aortic root, and the aortic valve (yellow arrow) with reimplantation of left and right coronary artery (LCA and RCA). Distal anastomosis is indicated by the white arrows.

Figure 6 shows the postoperative CTA examination and three-dimensional reconstruction with an excellent outcome of the Bentall procedure without nicking of the prosthesis.

## 4. Discussion

Minimally invasive cardiac surgery has been widely adopted worldwide and is showing a growing trend. Complex cardiac surgical procedures using the RAMT approach are increasingly performed in many cardiac surgery centers worldwide [7,8,9,10,11,12]. The aim of minimally invasive surgery is to avoid a complete sternotomy and its associated complications by reducing the morbidity and mortality [7,28,29]. Recently, minimally invasive surgeons are increasingly using the RAMT approach as an emerging technique to perform aortic valve surgery [7,8,9,10,11]. RAMT has shown better results in terms of blood loss and length of hospital stay than the sternotomy approach [11]. As surgeons employing minimally invasive approaches increase their experience, their interest in treating further complex pathologies, particularly in the AA or the aortic root via smaller surgical approaches, is also increasing. However, there is limited literature available specifically on treating aortic pathologies via the RAMT approach. Due to the technical challenges involved in aortic surgery, surgeons tend to approach these procedures with caution and prefer incisions that are closer to the aorta. In the past, various minimally invasive approaches, including upper partial sternotomy (T- or J-incision) as well as S-, L-, Z- and C-shaped mini-sternotomy, which have been used by aortic surgeons performing surgeries of the AA or the aortic root with good feasibility [16,17,18,19,20], are described in the literature. These techniques are promising and have already been shown to be as safe and effective as the conventional median sternotomy. All these minimally invasive techniques showed favorable postoperative outcomes, with reduced postoperative bleeding, reduced re-thoracotomy rate, faster extubation, reduced stay in ICU, lower risk of mediastinitis, faster discharge from hospital and recovery, and better esthetic results than median sternotomy [3,7,28,29].

To the best of our knowledge, this study is the largest in the field of aortic surgery using the full endoscopic RAMT approach with 3D visualization published to date. Conventional instruments and innovative suturing techniques were used without total circulatory arrest for distal anastomosis. Neither was rib resection performed, nor was the right internal mammary artery injured during the procedures.

LaPietra et al. first described surgical AVR with concomitant AAR via a right anterior thoracotomy approach with dislocation of the third or fourth costochondral cartilage. In their study, they included 20 patients with a mean age of 61 years and 80% male gender with excellent postoperative outcomes and short-term results. Distal anastomosis was performed under circulatory arrest in most of these patients (95%), and it is not known if video guidance was used [21].

In another retrospective study, Johnson et al. presented their surgical technique in seven selected patients who received an elective Bentall procedure using the RAMT approach. It was the first study describing the Bentall procedure using the RAMT approach. Also, they received satisfactory short-term results with favorable outcomes. Here, circulatory arrest was also used for suturing the distal anastomosis. Video guidance was used in all patients, and for performing the anastomosis, an automated suturing technology was applied in some cases [22].

Recently, Ji et al. presented their experience using the RAMT approach for performing the Bentall procedure on 15 selected patients. So far, it is the largest study presenting results about the Bentall procedure using the RAMT approach. In this small series, conventional instruments and suturing techniques were used without total circulatory arrest for distal anastomosis. The authors also did not disarticulate the ribs or injure the right internal mammary artery. They were able to show that a shortened length of intensive care unit as well as hospital stay did not coincide with in-hospital deaths, neurological complications, postoperative bleeding, or re-thoracotomy. In this study, the follow-up period was at least 6 months. During this time, all 15 patients were alive without reoperation [25].

So far, the longer procedure, as well as the CBP and aortic clamp time have been discussed as major disadvantages of the RAMT approach in the field of aortic surgery in comparison to sternotomy approaches. In our study, we have a relatively short CBP and aortic clamping time. Compared to the previously described studies, our times were significantly lower. The mean CPB time in this study was 94.9 min, which is much shorter than the time of 138.5 min in the case series by Ji et al. and 202.9 min in the series by Johnson et al. [22,25]. Similarly, the mean aortic cross-clamp time in our study was 63.8 min, which is also meaningfully shorter than the mean cross-clamp time of 95.0 min and 161.9 min reported in their study.

Compared to LaPietra et al. and Johnson et al., none of our patients needed circulatory arrest for distal anastomosis [21,22,23]. Our significantly shorter times could be justified by the high standardization of the operative steps, the high experience of the surgeon, the use of a 3D camera for better visualization, and the use of new innovations, like the RAM^®^ and Cor-Knot^®^ devices. All cases presented here were performed by a single surgeon with excellent surgical skills using minimally invasive surgery. In the study by Ji et al., it is not described if they used 3D visualization.

Our postoperative outcome showed comparable results to the results of LaPietra et al., Johnson et al., and Ji et al. [21,22,25]. We were able to show a lower ventilation time (5.8 h) compared to LaPietra et al., Johnson et al., and Ji et al. (11 h, 10.6 h, and 12.5 h, respectively). The ICU stay of our patients was 35.9 h, which was comparable to the ICU times of Johnson et al. (31.8 h) and Ji et al. (36 h) [21,22,25]. Our patients were discharged from the hospital after 7.8 days, which was slightly higher than the discharge time of Ji et al. (5.8 days) [25]. The re-thoracotomy rate in our study due to bleeding was zero and similar to LaPietra et al.’s results [21]. Cerebrovascular complications and 30-day mortality were also zero, which is in line with the aforementioned studies [21,22,25]. The summarized excellent results of the previous studies were able to be confirmed through our results.

During the median follow-up time of 18.2 months, which is much longer than the follow-up time of Ji et al. with 8.0 months, all our patients were alive and did not need re-interventions [25]. No follow-up data were collected in the studies of LaPietra et al. and Johnson et al. [21,22].

We were able to show that the totally endoscopic RAMT approach with 3D visualization, as a promising and emerging technique, can be safely transferred to the field of aortic surgery in selected patients. The high standardization of the operative steps by an expert team and the use of new surgical tools could make RAMT as fast and effective as sternotomy procedures but with the advantages of minimally invasive surgery resulting in less surgical trauma, faster extubation, shorter ICU and in-hospital stay, lower pain, faster recovery, and better cosmetic results. Reducing the length of hospital stay is an important aspect of resource use since ICU and hospital stays are the main determinants of cost after cardiac surgery [30].

In our center, the RAMT approach has become a common practice over the years, with an annual volume of over 50% of all surgical cases, and aortic surgical procedures using the RAMT approach are a potential option for selected patients. Patients benefit enormously from the RAMT approach. Together with the use of a totally endoscopic approach, smaller incisions that preserve the integrity and stability of the chest wall are achieved, while an excellent visualization of the surgical field and access to the aorta, aortic valve, and aortic root are able to be ensured. Therefore, the RAMT approach should be considered as a potential and promising operative approach for both isolated and complex AAR involving the aortic root and the aortic valve with a high degree of safety. The patients have a very short ventilation time and ICU stay and can be discharged very quickly, which leads to faster recovery. Moreover, the minimally invasive procedure offers a better cosmetic result of a smaller scar with a very low risk of wound infection, and mediastinitis. The perioperative safety and feasibility as well as the mid- and long-term results should be the goal of an emerging surgical technique, which should improve the existence of evidence-based results for a standard procedure.

## 5. Conclusions

The full endoscopic RAMT approach with 3D visualization is a safe, feasible, and promising technique, which can be transferred in the field of aortic surgery without compromising surgical quality, postoperative outcomes, or patient safety when the procedure is performed by an experienced team during minimally invasive surgery in a large-volume center.

## 6. Limitation

The main limitation of our study is its retrospective and non-randomized design, which may introduce selection bias. A further limitation is that a single surgeon with excellent expertise in minimally invasive surgery has performed all procedures. The lack of comparison with other approaches, like the upper or complete sternotomy, should also be considered as a limitation. The patient cohort is small. Further lager studies, especially in multicenter and randomized design, should be initiated to confirm the results of this study.

## Figures and Tables

**Table 1:** Patient preoperative baseline characteristics and preexisting conditions.

	Total *n* = 44	AAR *n* = 14	Wheat/Bentall *n* = 30	*p*-Value
**Baseline data**				
Age	61.4 ± 10.7	5.60 ± 12.3	63.1 ± 9.6	0.086
Male gender	28 (63.6%)	8 (57.1%)	20 (66.7%)	0.541
BMI	26.3 ± 3.9	26.7 ± 2.8	26.1 ± 4.3	0.296
**Aortic valve pathology and echocardiography data**				
Aortic regurgitation > I	14 (31.8%)	0 (0.0%)	14 (46.7%)	*<0.001*
*Degree of regurgitation*				
*0*	11 (25.0%)	7 (50.0%)	4 (13.3%)	*0.021*
*I*	19 (43.2%)	7 (50.0%)	12 (40.0%)	0.533
*II*	5 (11.4%)	0 (0.0%)	5 (16.7%)	0.161
*III*	9 (20.5%)	0 (0.0%)	9 (30.0%)	*0.040*
Aortic stenosis	16 (36.4%)	0 (0.0%)	16 (53.3%)	*<0.001*
*Severity of stenosis*				
*AVA (cm^2^)*	0.9	-	0.9	-
*Pmean (mmHg)*	42.0	-	42.0	-
Bicuspid aortic valve	21 (47.7%)	5 (35.7%)	16 (53.3%)	0.276
Ejection fraction (%)	59.9 ± 6.2	62.5 ± 4.2	58.7 ± 6.6	0.059
**Previous medical history**				
Hypertension	41 (93.2%)	13 (92.9%)	28 (93.3%)	0.953
Hyperlipidemia	15 (34.1%)	3 (21.4%)	12 (40.0%)	0.194
Nicotine abuse	7 (15.9%)	3 (21.4%)	4 (13.3%)	0.396
Diabetes mellitus	5 (11.4%)	2 (14.3%)	3 (10.0%)	0.515
Coronary artery disease	8 (18.2%)	1 (7.1%)	7 (23.3%)	0.194
Chronic obstructive pulmonary disease	2 (4.5%)	1 (7.1%)	1 (3.3%)	0.540
Peripheral arterial vascular disease	3 (6.8%)	0 (0.0%)	3 (10.0%)	0.307
Cerebrovascular disease	3 (6.8%)	0 (0.0%)	3 (10.0%)	0.307
Previous stroke	4 (9.1%)	1 (7.1%)	3 (10.0%)	0.621
Atrial fibrillation	7 (15.9%)	0 (0.0%)	7 (23.3%)	0.053
Vitamin K antagonist or NOAC therapy	7 (15.9%)	0 (0.0%)	7 (23.3%)	0.053
Chronic kidney failure	2 (4.5%)	0 (0.0%)	2 (6.7%)	0.460
Preoperative Creatinine (mg/dL)	0.95 ± 0.25	0.87 ± 0.18	0.98 ± 0.27	0.094
Bovine arch	3 (6.8%)	1 (7.1%)	2 (6.7%)	0.693
**Clinical presentation**				
NYHA I	5 (11.4%)	5 (35.7%)	0 (0.0%)	*0.002*
NYHA II	17 (38.6%)	4 (28.6%)	13 (43.3%)	0.275
NYHA III	22 (50.0%)	5 (35.7%)	17 (56.7%)	0.195
**Preoperative CT measurement**				
Diameter of the Aorta				
*Annulus fibrosus (mm)*	26.3 ± 1.9	26.0 ± 1.9	26.7 ± 2.2	0.655
*Sinus valsalva (mm)*	41.8 ± 5.1	35.7 ± 6.2	42.3 ± 4.6	0.524
*Sino-tubular junction (mm)*	36.9 ± 6.1	36.2 ± 8.1	37.2 ± 5.3	0.555
*Ascending aorta (mm)*	51.2 ± 4.6	52.9 ± 5.3	50.4 ± 4.1	0.178
*Proximal aortic arch*	36.1 ± 2.7	37.3 ± 2.9	35.5 ± 2.4	0.119

Abbreviations: AAR: supracoronary replacement of the ascending aorta; AVA: aortic valve area; BMI: body mass index; NYHA: New York Heart Association; Pmean: mean aortic gradient. Values are expressed as mean standard deviation or as number and percentage (in brackets). Significant results are displayed in italics.

**Table 2:** Intra- and postoperative outcome and follow-up details.

	Total *n* = 44	AAR *n* = 14	Wheat/Bentall *n* = 30	*p*-Value
**Intraoperative data**				
Time to skin closure	140.6 ± 33.2	121.0 ± 27.1	149.8 ± 32.2	*0.006*
CPB time in min	94.9 ± 32.5	68.6 ± 19.7	107.2 ± 30.0	*<0.001*
Aortic X-clamp time in min	63.8 ± 25.9	42.9 ± 19.4	73.5 ± 22.7	*<0.001*
min body temperature during CBP in °C	35.5 ± 0.9	35.3 ± 0.9	35.6 ± 0.9	0.342
**Intraoperative transfusion**				
RBC	0.4 ± 1.0	0.1 ± 0.3	0.5 ± 1.2	0.324
FFP	0.2 ± 1.1	0.0 ± 0.0	0.4 ± 1.3	0.321
PC	0.3 ± 0.6	0.1 ± 0.3	0.4 ± 0.6	0.168
**Postoperative outcome**				
Bleeding	0 (0.0%)	0 (0.0%)	0 (0.0%)	-
Pericardial effusion	0 (0.0%)	0 (0.0%)	0 (0.0%)	-
Re-thoracotomy	0 (0.0%)	0 (0.0%)	0 (0.0%)	-
Myocardial infarction	0 (0.0%)	0 (0.0%)	0 (0.0%)	-
Conversion to sternotomy	0 (0.0%)	0 (0.0%)	0 (0.0%)	-
Renal failure	0 (0.0%)	0 (0.0%)	0 (0.0%)	-
Stroke	0 (0.0%)	0 (0.0%)	0 (0.0%)	-
Delirium	4 (9.1%)	1 (7.1%)	3 (10.0%)	0.621
Atrial fibrillation	9 (20.5%)	0 (0.0%)	9 (30.0%)	*0.020*
Pacemaker implantation	0 (0.0%)	0 (0.0%)	0 (0.0%)	-
Wound infection	0 (0.0%)	0 (0.0%)	0 (0.0%)	-
*Mortality*				
*In-hospital mortality*	0 (0.0%)	0 (0.0%)	0 (0.0%)	-
*30-day mortality*	0 (0.0%)	0 (0.0%)	0 (0.0%)	-
*1-year mortality*	0 (0.0%)	0 (0.0%)	0 (0.0%)	-
Re-intervention	0 (0.0%)	0 (0.0%)	0 (0.0%)	-
Actual status alive	44 (100.0%)	14 (100.0%)	30 (100.0%)	-
**Postoperative transfusion**				
RBC	0.8 ± 1.2	0.7 ± 0.9	0.8 ± 1.3	0.970
FFP	0.4 ± 1.3	0.0 ± 0.0	0.5 ± 1.5	0.217
PC	0.1 ± 0.3	0.0 ± 0.0	0.1 ± 0.4	0.488
**Ventilation time in h**	5.8 ± 7.6	6.1 ± 7.2	5.7 ± 7.9	0.710
**ICU time in h**	35.9 ± 23.5	36.4 ± 29.0	39.0 ± 27.3	0.164
**Length of hospital stay in d**	7.8 ± 3.0	8.5 ± 3.7	7.4 ± 2.6	0.321

Abbreviations: AAR: supracoronary replacement of the ascending aorta; CPB: cardiopulmonary bypass; FFP: fresh frozen plasma; ICU: intensive care unit; PC: platelet count; postop, postoperative; RBC: red blood cell concentrate. Values are expressed as mean standard deviation or as number and percentage (in brackets). Significant results are displayed in italics.

## Data Availability

The data presented in this study are available on request from the corresponding author.

## Supplementary Materials

The following supporting information can be downloaded at https://www.mdpi.com/article/10.3390/jcm13092648/s1, Video S1: Minimally invasive aortic valve and ascending aorta replacement (https://zenodo.org/records/10946359), Table S1: Surgeries performed in participating centers.
