# Supplementary material for: Totally Endoscopic Replacement of the Ascending Aorta and the Aortic Root including the Aortic Valve via Right Mini-Thoracotomy: A Multicenter Study
Source: J Clin Med. 2024 Apr 30;13(9):2648. doi: 10.3390/jcm13092648 (PMC11084888; doi:10.3390/jcm13092648)
Supplement: Supplementary file 1 [file jcm-13-02648-s001.zip › jcm-2979742-supplementary.pdf]

Table S1. Surgeries performed in participating centers.

	Total n=44	Bonn n=30	Siegburg n=10	Wuppertal n=4
**Type of Surgery**				
Supracoronary AAR	14 (31.8%)	9 (30.0%)	5 (50.0%)	0 (0.0%)
Wheat-Procedure	21 47.7%)	14 (46.7%)	4 (40.0%)	3 (75.0%)
Bentall-Procedure	8 (18.2%)	7 (23.3%)	1 (10.0%)	1 (25.0%)

Abbreviations: AAR: Replacement of the ascending aorta.
